# Real-world patient-reported outcomes of women receiving initial endocrine-based therapy for HR+/HER2− advanced breast cancer in five European countries

**DOI:** 10.1186/s12885-020-07294-2

**Published:** 2020-09-07

**Authors:** Alison Davie, Gebra Cuyun Carter, Alex Rider, James Pike, Katie Lewis, Abigail Bailey, Gregory L. Price, Francois Ringeisen, Xavier Pivot

**Affiliations:** 1 Eli Lilly and Co Ltd, Windlesham, Surrey, GU20 6PH UK; 2 Eli Lilly and Co, Indianapolis, IN 46204 USA; 3 Adelphi Real World, Bollington, Macclesfield, Cheshire, SK10 5JB UK; 4 Eli Lilly SA, Vernier, Geneva, Switzerland; 5 Paul Strauss Cancer Center, Strasbourg, France

**Keywords:** Advanced breast cancer, Health-related quality of life, Real-world evidence, Patient-reported outcomes

## Abstract

**Background:**

Endocrine therapy (ET)-based regimens are the mainstay of treatment for patients with hormone receptor-positive/human epidermal growth factor receptor 2-negative (HR+/HER2−) advanced breast cancer. With the introduction of new treatment classes, it is important to examine patient symptoms and health-related quality of life (HRQoL) at the start of this changing therapeutic landscape. This real-world study describes the patient-reported outcomes (PROs) of women with HR+/HER2− advanced breast cancer receiving ET-based regimens who were naïve to systemic treatment in the advanced setting across five European countries (EU5).

**Methods:**

Data were collected between March and July 2017 from surveyed oncologists and their patients at a single time point using the multinational Adelphi Advanced Breast Cancer Disease Specific Programme™. Patients completed PRO questionnaires on HRQoL (EORTC QLQ-C30), pain severity and interference, and work and activity impairment. A multiple linear regression model explored factors associated with HRQoL.

**Results:**

Across EU5, 226 physicians provided data on 781 women with HR+/HER2− advanced breast cancer taking their first ET-based regimen for advanced disease, of whom 252 provided PRO data. This subset had a mean age of 67.1 years, 94% were postmenopausal, 89% were diagnosed with advanced breast cancer at initial presentation, 79% had stage IV disease (66% of these patients had bone metastases and 38% had visceral metastases, including 18% with liver metastases) and 77% were on endocrine-only therapy as their initial treatment for advanced disease. The mean EORTC QLQ-C30 global health score (50.9) was worse than the reference value for patients with advanced breast cancer (60.2). Fatigue, pain, and insomnia were the most severe symptoms, and mean functioning scores were also worse than reference values. “Worst pain” and “pain interference” were moderate/severe for 42 and 80% of patients. Mean activity impairment was 44%, and greater activity impairment was associated with poorer HRQoL.

**Conclusions:**

Despite receiving first-line ET-based regimens for advanced disease, these women had a poor HRQoL and high levels of symptoms, pain, pain interference and activity impairment. New treatments that maintain a stable disease state and reduce activity impairment may have a positive effect on the HRQoL of those living with advanced breast cancer.

## Background

Breast cancer is the most common cancer in women across Europe and worldwide [1, 2]. In 2018, it was estimated there would be 523,000 new cases of breast cancer in women in Europe and that 138,000 women would die from breast cancer in the same year [1]. Advanced breast cancer is defined as locally advanced (stage IIIb/c) or metastatic (stage IV). An estimated 5–10% of all breast cancer patients have metastatic disease at initial presentation (de novo) [3], whereas approximately 30% of patients diagnosed with early-stage disease will progress to develop metastatic disease [4]. Hormone receptor-positive/human epidermal growth factor receptor 2-negative (HR+/HER2−) is the most common subtype of advanced breast cancer (66%) and incurs a median overall survival of 24.8 months after diagnosis of distant metastases [5, 6]. Patients with the HR+/HER2− subtype have a more favorable prognosis than those with other breast cancer subtypes [5, 7].

Treatment for HR+/HER2− advanced breast cancer is primarily palliative and aims to delay disease progression, prolong life, reduce disease- and/or treatment-related symptoms, improve daily functioning, and maintain/improve health-related quality of life (HRQoL) [8]. However, outcomes such as survival and HRQoL still need to be improved for patients with HR+/HER2− advanced breast cancer [6]. Patient-reported outcome (PRO) measures are often used in randomized clinical trials to evaluate symptoms and HRQoL, but there are concerns regarding the heterogeneity of the methodology used, analysis, and quality of reporting [9–11]. It is important to capture real-world symptom and HRQoL data from patients living with advanced breast cancer, as patients enrolled in clinical trials are more homogeneous than the heterogeneous population in the real world.

PRO data on patients with advanced breast cancer in the real-world setting are limited, especially in those with HR+/HER2− disease [12–17]. Evidence is also lacking from European settings. Much of the existing data come from Canada and the United States (US), which may differ from Europe with respect to healthcare systems and routine treatment practices [18].

The 4th European School of Oncology–European Society for Medical Oncology (ESO–ESMO) international consensus guidelines for advanced breast cancer recommend endocrine therapy (ET)-based regimens as the preferred first-line treatment option for women with HR+/HER− advanced breast cancer [8]. Because of the heterogeneous and incurable nature of the disease, most patients with HR+ advanced breast cancer will develop endocrine resistance, leading to relapse and disease progression. However, targeted therapies, such as inhibitors of cyclin-dependent kinases (CDK) 4 and 6 or the mammalian target of rapamycin (mTOR), can now be used in combination with ET to improve outcomes and delay the development of endocrine resistance [8]. This is a rapidly evolving therapeutic field, and three CDK4 & 6 inhibitors (palbociclib, ribociclib, and abemaciclib) have now been approved in the European Union (EU) for use in combination with ET as first-line advanced therapy for those naïve to systemic therapy in this setting. With the introduction of this new drug class, changes in treatment paradigms can be expected, with the goal of extending patients’ lives and maintaining/improving their HRQoL. Presently, questions to be addressed include the optimal initial therapy for advanced disease and the subsequent sequence of treatments for delaying disease progression and improving/maintaining the HRQoL of those living with HR+/HER− advanced breast cancer.

The aim of this study was to describe the characteristics, HRQoL, and symptoms of women with HR+/HER2− advanced breast cancer receiving their first ET-based regimen for advanced disease in real-world clinical practice across five European countries (EU5), using PRO data collected as part of the Adelphi Advanced Breast Cancer Disease Specific Programme™ (DSP). The survey was conducted in 2017 at the start of the change in the therapeutic landscape for such patients.

## Methods

### Study design, study participants, and data collection

The Adelphi Advanced Breast Cancer DSP is a large, multinational, real-world, point-in-time, patient record-based survey in the clinical practice setting that describes current disease management, disease-burden impact, and associated treatment effects. The DSP methodology has been described previously [19]. It includes physicians and patients across a range of care settings: comprehensive cancer centers, university hospitals, general hospitals, community hospitals, and physician offices. Patients were excluded from the DSP if they were participating in a clinical trial. Data for this Advanced Breast Cancer DSP were collected between March and July 2017 at a single time point for each participating patient and physician in France, Germany, Italy, Spain, and the United Kingdom (UK).

Oncologists were identified from publicly available lists of healthcare professionals, then selected at random (to avoid bias) and invited to participate in the DSP. Recruitment aimed to cover the geographical spread of each participating country. To be eligible for inclusion, oncologists had to have consultations with at least three patients with advanced breast cancer in a typical month and be personally responsible for the management of and treatment decisions for these patients.

Physicians completed patient record forms (PRFs) for the next seven consenting adult female patients (aged ≥18 years) with a confirmed diagnosis of advanced breast cancer (any hormone receptor status) and who were receiving active drug treatment for their advanced disease at the time of survey. To oversample the patient population of interest, PRFs were then completed for the next three patients who presented with HR+/HER2− advanced breast cancer. PRFs recorded patient demographics, clinical characteristics, and details of prior and current treatment.

At the time of consultation, each patient with a PRF was invited to complete an optional patient self-completion form (PSC) containing questions on their education, employment status, input to treatment decisions, and current disease status. Patients were also asked to complete several PRO questionnaires that assessed their HRQoL, general health status, work productivity and activity, pain, and pain interference at the time of the most recent consultation.

### PRO questionnaires

All PRO questionnaires were administered in the native language of each country.

HRQoL was assessed using the European Organization for Research and Treatment of Cancer Quality of Life Questionnaire–Core 30 (EORTC QLQ-C30, version 3.0) [20] and the complementary breast cancer-specific module (QLQ-BR23, version 1.0) [21]. The EORTC QLQ-C30 is a standardized, validated questionnaire that comprises 30 items divided among five functional scales (physical, role, cognitive, emotional, and social), nine symptom scales (fatigue, nausea/vomiting, pain, dyspnea, insomnia, appetite loss, constipation, diarrhea, financial difficulties), and a global health/QoL scale. QLQ-BR23 comprises 23 items that assess symptoms and treatment side effects across four functional scales (body image, sexual functioning, sexual enjoyment, future perspective) and four symptom scales (systemic therapy side effects, breast symptoms, arm symptoms, and upset by hair loss). Each item was rated on a scale ranging from 1 to 4, except for the global health/QoL scale, which ranged from 1 to 7. A scoring algorithm was used to convert the responses to a scale ranging from 0 to 100 [22]. For the functional and global health/QoL scales, a higher score represents a better level of functioning/QoL. For the symptom scales, a higher score represents worse symptom severity.

General health status was assessed using the EuroQol 5-dimensions 3-level questionnaire (EQ-5D-3L), which comprises five dimensions (mobility, self-care, usual activities, pain/discomfort, anxiety/depression) and a 20-cm visual analogue scale (EQ-VAS) [23]. Each dimension is scored from 1 (no problems) to 3 (severe problems) and country-specific algorithms were applied to these scores to generate a single health utility index, where a score of 1 indicates perfect health, 0 indicates death, and < 0 indicates worse than death [24]. The EQ-VAS provides a score ranging from 0 to 100, with higher scores indicating a better general health status.

Pain and the degree to which it interferes with a patient’s daily life was assessed using a modified version of the Brief Pain Inventory (BPI) [25]. The BPI comprises four items related to pain severity (worst pain in last 24 h, least pain in last 24 h, average pain in last 24 h, pain right now) and seven items related to interference by pain in the past 24 h (general activity, mood, walking ability, normal walk, relations with other people, sleep, enjoyment of life). Each item was rated from 0 to 10, and a single pain interference score was calculated as the average of the seven pain interference item scores, with higher scores reflecting worse pain severity or pain interference.

The Work Productivity and Activity Impairment Questionnaire (WPAI) assesses impairment at work and in non-work activities [26]. The Specific Health Problem version (WPAI:SHP) was used, which comprises six items related to productivity over the past 7 days and provides four scores, expressed as percentages: work time missed (absenteeism), impairment while working (presenteeism), overall work impairment (work productivity loss), and total activity impairment. Higher percentages indicate greater work/activity impairment due to breast cancer.

All PRO questionnaires were administered at the time of PSC completion, and the number of responses vary as PSCs were completed voluntarily.

### Analyses

The following patient cohort was identified from the total patient population in the Advanced Breast Cancer DSP: EU5 patients with HR+/HER2− advanced breast cancer currently receiving an ET-based regimen as their initial treatment for advanced disease at the time of data collection. These HR+/HER2− patients had either advanced breast cancer (stage IIb/IIIc/IV) at diagnosis or had been diagnosed with early-stage disease (stage I/II/IIIa) and initiated their first-line ET-based regimen for advanced breast cancer more than 12 months after completing (neo) adjuvant ET because of disease recurrence. Thus, the EU5 patients in our analyses were considered endocrine responsive. Treatments used at the time of data collection were grouped as follows: Endocrine only, Endocrine + targeted therapy, Endocrine + chemotherapy, Endocrine + chemotherapy + targeted therapy, Endocrine + other (unspecified). Targeted therapies included CDK4 & 6 inhibitors, everolimus, bevacizumab (used in combination with chemotherapy) and others (including HER2+ treatments reported for three patients).

Physician characteristics, patient characteristics, and PRO data were analyzed using descriptive statistics and reported by country and for the five European countries pooled (EU5). Means and standard deviations (SDs) and/or medians and interquartile ranges (IQRs) were calculated for continuous variables, whereas frequency counts and percentages were calculated for categorical variables. BPI “worst pain” item severity scores were grouped as none (0), mild (1–4), moderate (5–6), or severe (7–10) [27, 28], and BPI “pain interference” scores (average of all seven items) were grouped as mild (0–1), moderate (2–5), or severe (6–10) [29] and are presented as frequency distributions. The statistical significance of differences between countries was analyzed using Pearson’s Chi-squared tests or Fisher’s exact test (for categorical variables) or Student’s t-test, analysis of variance, or Kruskal–Wallis tests (for numerical variables).

Because the data analysis showed that German women accounted for 40% of the PRO subset and had some differences in characteristics from the women from the other four countries combined (EU4), we performed post-hoc analyses to compare the German cohort with the EU4 cohort.

A multiple linear regression model was used to explore associations between covariates and HRQoL as assessed using the EORTC QLQ-C30 global health/QoL scale score. Covariates included in the model were country, patient age, total number of comorbidities, current performance status (PS) as assessed by Eastern Cooperative Oncology Group (ECOG) score (PS = 0, 1, 2–4), physician perception of current disease status (stable, progressing, responding to treatment), liver metastases (absent, present), number of current metastases (excluding liver metastases), patient-reported pain (EQ-5D pain/discomfort domain), ongoing ET treatment duration (weeks), and activity impairment (percent activity impairment on WPAI) at the single time point when the data were collected for each patient. Each variable was selected according to expert knowledge of the disease and with the goal of minimizing multicollinearity. Standard errors were adjusted using a robust clustered sandwich estimator of variance to allow for intragroup correlation within reporting physician, relaxing the usual requirement that the observations be independent [30–32]. That is, the patients were considered independent across different physicians but not necessarily when they had the same physician. Answers were dependent on patient participation, and missing data were not imputed. The number of patients included in each analysis is reported.

A sensitivity analysis was performed to examine the multi-level nature of the data in a different way. We used a three-level linear fixed-effects model with random intercepts at both the country and physician level to account for correlation within countries and physicians. The dependent variable was the EORTC QLQ-C30 global health/QoL scale score and the fixed-effects covariates were the same as those used in the linear regression model. Descriptive statistics were derived using IBM SPSS Data Collection Survey Reporter version 7 software (SPSS [Hong Kong] Ltd., Quarry Bay, Hong Kong). All analyses that required statistical comparisons were conducted using STATA statistical software version 15.1 (StataCorp LLC, College Station, TX, USA).

### Ethical considerations

The Advanced Breast Cancer DSP was undertaken in line with European Pharmaceutical Marketing Research Association guidelines [33], adhering to the International Chamber of Commerce/European Society for Opinion and Marketing Research (ICC/ESOMAR) International Code on observational research [34], and was reviewed and approved by the Freiburg Ethics Commission International (FEKI), an institutional review board. All patients included in the DSP provided written informed consent to participate. All data were anonymized and aggregated prior to receipt by Adelphi Real World for analysis.

## Results

### Analysis cohort

Overall, 226 physicians across the EU5 provided data on the analysis cohort (i.e., patients with HR+/HER2− advanced breast cancer currently receiving an ET-based regimen as initial treatment for advanced disease). Across the EU5, 781 patients were receiving their initial ET-based regimen for advanced disease, and 252 of these patients completed PSCs. Additional file 1 gives the physician and patient sample sizes (Table S1) and Additional file 2 summarizes physician characteristics (Table S2) by country and overall (EU5).

### Patient characteristics

The characteristics of the 252 women with HR+/HER2− advanced breast cancer currently receiving an ET-based regimen as their initial treatment for advanced disease and who provided PRO data are summarized in Table 1 by country and for EU5 (data taken from PRF). Across the EU5, these women had a mean (SD) age of 67.1 (10.8) years, and 94% were postmenopausal. The majority of women who provided PRO data (*n* = 223; 89%) were diagnosed with advanced disease (stage IIIb/IIIc/IV), and 200 women (79%) had stage IV disease at the time of data collection: 66% of these patients had bone metastases and 38% had visceral metastases (24% had lung metastases, 18% had liver metastases). Current breast cancer status was reported by the physician as stable for 58% of patients, and 35% were considered as responding to treatment. At the time of data collection, 77% of patients were on endocrine-only treatment for advanced disease, and patients had been on their current treatment regimen for a mean (SD) of 6.7 (8.6) months (Table 1). A total of 43 patients (17%) received targeted therapy in combination with ET, of whom 17 received a CDK4 & 6 inhibitor (palbociclib), representing 7% of the study cohort providing PRO data. For EU5 patients who were initially diagnosed with early stage breast cancer and had disease recurrence (*n* = 29), the mean (SD) time since the end of adjuvant treatment and start of the first-line ET-based regimen for advanced disease (i.e., treatment-free interval) was 38.8 (27.3) months.

**Table 1:** Key characteristics of women with HR+/HER2− advanced breast cancer currently receiving initial endocrine-based therapy for advanced disease: subset of patients providing PRO data

Characteristics	France (*N* = 69)	Germany (*N* = 100)	Italy (*N* = 18)	Spain (*N* = 48)	UK (*N* = 17)	EU5 (*N* = 252)	Comparison between EU5 countries *p*-value [test used]	Germany vs. EU4 *p*-value [test used]
Age,^a^ years, mean (SD)	65.2 (11.4)	69.0 (9.3)	61.0 (11.5)	64.1 (11.2)	68.1 (11.9)	67.1 (10.8)	**< 0.001** [AN]	0.369 [TT]
BMI, kg/m^2^, mean (SD)	24.0 (3.4)	24.3 (4.1)	23.8 (3.5)	25.5 (3.6)	24.6 (3.1)	24.4 (3.7)	0.281 [AN]	0.125 [TT]
Ethnicity, *n* (%)							0.343 [CH]	0.801 [CH]
White/Caucasian	61 (88)	94 (94)	18 (100)	46 (96)	16 (94)	235 (93)		
Other^b^	8 (12)	6 (6)	0	2 (4)	1 (6)	17 (7)		
Employment status, *n* (%)	(*n* = 68)	(*n* = 98)	(*n* = 18)	(*n* = 47)	(*n* = 16)	(*n* = 247)	**0.015** [CH]	0.932 [CH]
Retired/homemaker/unemployed	60 (88)	66 (67)	16 (89)	41 (87)	15 (94)	198 (80)		
Employed FT/PT	5 (7)	17 (17)	2 (11)	4 (9)	1 (6)	29 (12)		
Long-term sick leave (FT/PT)	3 (4)	15 (15)	0	2 (4)	0	20 (8)		
Current^c^ ECOG status^d^, *n* (%)	(*n* = 69)	(*n* = 100)	(*n* = 18)	(*n* = 48)	(*n* = 17)	(*n* = 252)	0.065 [CH]	0.055 [CH]
0	11 (16)	36 (26)	9 (50)	13 (27)	8 (47)	77 (31)		
1	45 (65)	42 (42)	8 (44)	26 (54)	7 (41)	128 (51)		
2	10 (15)	18 (18)	1 (6)	9 (19)	2 (12)	40 (16)		
3–5	3 (4)	4 (4)	0	0	0	7 (3)		
Current^c^ menopausal status, *n* (%)							0.937 [CH]	0.847 [FE]
Pre−/perimenopausal	3 (4)	6 (6)	1 (6)	4 (8)	1 (6)	15 (6)		
Postmenopausal^e^	66 (96)	94 (94)	17 (94)	44 (92)	16 (94)	237 (94)		
Number of current^c^ metastases sites^f^, *n* (%)	(*n* = 65)	(*n* = 60)	(*n* = 15)	(*n* = 44)	(*n* = 16)	(*n* = 200)	0.101 [AN]	**0.037** [TT]
1	49 (75)	35 (58)	9 (60)	21 (48)	13 (81)	127 (64)		
2	14 (22)	15 (25)	5 (33)	16 (36)	3 (19)	53 (27)		
3	2 (3)	6 (10)	1 (7)	7 (16)	0	16 (8)		
4–5	0	4 (7)	0	0	0	4 (2)		
Site of current^c^ metastases^f^, *n* (%)	(*n* = 65)	(*n* = 60)	(*n* = 15)	(*n* = 44)	(*n* = 16)	(*n* = 200)	[CH]	[FE]
Bone only	35 (54)	13 (22)	7 (47)	17 (39)	11 (69)	83 (42)	**< 0.001**	0.174
Bone	45 (69)	23 (38)	12 (80)	38 (86)	13 (81)	131 (66)	**< 0.001**	0.199
Liver	10 (15)	18 (30)	2 (13)	4 (9)	1 (6)	35 (18)	**0.034**	0.667
Lung	11 (17)	17 (28)	2 (13)	17 (39)	1 (6)	48 (24)	**0.024**	**0.012**
Lymph node involvement	12 (19)	39 (65)	6 (40)	11 (25)	3 (19)	71 (36)	**< 0.001**	0.323
Visceral^g^	22 (34)	26 (43)	4 (27)	21 (48)	3 (19)	76 (38)	0.178	0.144
Other (including brain & pancreas)	4 (6)	3 (5)	0	4 (9)	1 (6)	12 (6)	0.770	0.669
Breast cancer stage at diagnosis, *n* (%)							0.108 [CH]	0.913 [FE]
Stage IIIb/IIIc/IV	57 (83)	95 (95)	16 (89)	41 (85)	14 (82)	223 (89)		
Stage I/II/IIIa	12 (17)	5 (5)	2 (11)	7 (15)	3 (18)	29 (12)		
Current^c^ breast cancer stage, *n* (%)							**< 0.001** [CH]	**< 0.001** [CH]
Stage IIIb	0 (0)	2 (2)	0 (0)	1 (2)	0 (0)	3 (1)		
Stage IIIc	4 (6)	38 (38)	3 (17)	3 (6)	1 (6)	49 (19)		
Stage IV	65 (94)	60 (60)	15 (83)	44 (92)	16 (94)	200 (79)		
Current^c^ number of symptoms, mean (SD)	1.7 (1.1)	1.1 (1.4)	1.7 (1.3)	1.6 (1.0)	1.2 (0.7)	1.4 (1.3)	**0.022** [AN]	**0.002** [TT]
Charlson comorbidity index score, mean (SD)	2.3 (0.7)	2.3 (0.8)	2.2 (0.5)	2.2 (0.7)	2.2 (0.4)	2.3 (0.7)	0.903 [AN]	0.463 [TT]
Current^c^ disease status^h^, *n* (%)							**0.015** [CH]	0.208 [CH]
Stable	28 (41)	69 (69)	12 (67)	28 (58)	10 (59)	147 (58)		
Tumor responding to treatment	35 (51)	23 (23)	5 (28)	19 (40)	7 (41)	89 (35)		
Progressing	6 (9)	8 (8)	1 (6)	1 (2)	0	16 (6)		
Current^c^ treatment class^i^, *n* (%)							0.139 [CH]	0.273 [CH]
Endocrine only	50 (73)	70 (70)	14 (78)	43 (90)	16 (94)	193 (77)		
Endocrine + targeted^j^	10 (15)	13 (13)	4 (22)	5 (10)	1 (6)	33 (13)		
Endocrine + chemotherapy	4 (6)	12 (12)	0	0	0	16 (6)		
Endocrine + chemotherapy + targeted	4 (6)	5 (5)	0	0	0	9 (4)		
Endocrine + other	1 (1)	0	0	0	0	1 (< 1)		
Current ET-based regimen duration^k^ (ongoing), months	(*n* = 69)	(*n* = 100)	(*n* = 18)	(*n* = 48)	(*n* = 17)	(*n* = 252)	0.412 [AN]	0.117 [TT]
Mean (SD)	6.0 (5.9)	7.0 (10.9)	5.5 (7.9)	8.2 (8.2)	4.0 (3.5)	6.7 (8.6)		
Median	4.3	4.2	2.9	6.9	3.0	4.1		
IQR	2.1, 7.5	1.9, 8.3	1.6, 6.3	1.9, 12.0	1.7, 4.4	2.0, 8.6		
TFI^l^, months	(*n* = 12)	(*n* = 5)	(*n* = 2)	(*n* = 7)	(*n* = 3)	(*n* = 29)	0.253 [AN]	0.479 [TT]
Mean (SD)	29.3 (24.9)	57.8 (25.1)	54.6 (2.2)	32.3 (21.9)	49.6 (47.9)	38.8 (27.3)		
Median	19.7	58.0	54.6	20.2	27.4	27.9		
IQR	17.3, 32.4	39.5, 73.4	52.0, 56.1	15.7, 57.1	16.8, 104.5	18.0, 56.1		

Data taken from PRF

*AN* analysis of variance, *BMI* body mass index, *CH* Pearson’s Chi-squared test, *ECOG* Eastern Cooperative Oncology Group, *ET* endocrine therapy, *EU5* European Union 5, *EU4* European Union 4 (France, Italy, Spain and UK), *FE* Fisher’s exact test, *FT* full time, *HER2+* human epidermal growth factor receptor 2-positive, *HR+/HER2−* hormone receptor-positive/human epidermal growth factor receptor 2-negative, *IQR* interquartile range, *PRF* patient record form, *PT* part time, *SD* standard deviation, *TFI* treatment-free interval, *TT* Students t-test, *UK* United Kingdom

^a^Patients reported to be ≥90 years of age were assumed to be 90 years of age for the purposes of this calculation

^b^Includes Afro-Caribbean, Hispanic/Latino, mixed race, Asian-other, Asian-Indian subcontinent, Middle Eastern and Chinese

^c^Current = time of data collection

^d^0 = Fully active, able to carry on all pre-disease performance without restriction; 1 = Restricted in physically strenuous activity but ambulatory and able to carry out light work; 2 = Ambulatory and capable of all self-care but unable to carry out work activities. Up and about more than 50% of waking; 3 = Capable of only limited self-care, confined to bed or chair more than 50% of waking hours; 4 = Completely disabled. Cannot carry out any self-care. Confined to bed or chair; two patients were not assessed

^e^Including natural, medically induced ovary suppression and ablation

^f^Based on patients currently at stage IV

^g^Visceral metastases = presence of brain, liver, lungs, pancreas, and other (including pleural) metastases

^h^Physician reported

^i^Simultaneous treatments

^j^Targeted therapy for any patient included palbociclib (*n* = 17), everolimus (*n* = 14), bevacizumab (*n* = 9), and other (*n* = 3)

^k^All patients were currently still on first-line therapy; duration does not reflect how long patients stay on a first-line regimen

^l^Treatment-free interval between adjuvant therapy and first-line advanced ET for the patients with Stage I/II/IIIa disease at diagnosis

The characteristics of the overall patient cohort (EU5, *n* = 781) are given in Additional file 3 Table S3 where significant between-country differences were seen for many patient characteristics. To investigate this further, we compared the characteristics of the German cohort (accounting for 25% of the full sample and 40% of PRO subset) with patients in the other four countries combined (EU4). At the time of data collection, the PRO subset of German women had fewer symptoms and were less likely to have stage IV breast cancer, but were more likely to have multiple metastatic sites and lung metastases present than the EU4 subset providing PRO (Table 1). Also, the ECOG score approached statistical significance, with German women showing a worse performance status than the EU4 women.

### Patient-reported HRQoL and health status

Table 2 shows that the EORTC QLQ-C30 mean global health/QoL scale score for the subset of EU5 patients who provided PRO data was 50.9, which was significantly lower (worse) than the mean reference value of 60.2 for patients with recurrent/metastatic breast cancer [35] and the mean European normative score of 72.3 for women in the general population aged 60–69 years [36]. Likewise, the mean scores for the five EORTC QLQ-C30 functional scales for EU5 were significantly lower (worse) than both the normative and the reference values for patients with recurrent/metastatic breast cancer (Table 2). The EORTC QLQ-C30 mean symptom scale scores show that fatigue, pain, and insomnia were the most severe symptoms, consistent with the order of severity of the reference values for patients with recurrent/metastatic breast cancer (Table 2). The subset of EU5 women providing PRO data had significantly higher (worse) mean symptom scores for fatigue, nausea/vomiting, pain, dyspnea, appetite loss and diarrhea, compared with the reference values. The median scores for the global health/QoL, functional and symptom scales were similar to their respective means (data not shown). The country-specific and EU4 EORTC QLQ-C30 scores are given in Additional file 4: Table S4. Each scale score was significantly worse for German women vs EU4 women (*p* < 0.001, Mann-Whitney test). Each of the mean EORTC QLQ-C30 scores for Germany also differed significantly from the mean reference value for women with advanced breast cancer (*p* < 0.001, Student’s t-test).

**Table 2:** Patient-reported EORTC QLQ-C30 scores (subset of patients who provided PRO data)^a^

	**EU5**		***Reference values for advanced breast cancer***^***b***^	***Difference in means (reference value − EU5)***	***Normative values***^***c***^	***Difference in means (normative value − EU5)***
	***n***	**Mean (SD)**	***Mean (SD)***		***Mean (SD)***	
Global health/QoL scale	249	50.9 (24.7)	60.2 (25.5)	9.3**	72.3 (20.5)	21.4**
Functional scales						
Physical functioning	245	63.5 (24.6)	81.6 (18.7)	18.1**	85.0 (15.9)	21.5**
Role functioning	244	59.9 (27.2)	67.4 (31.1)	7.5**	84.0 (22.4)	24.1**
Emotional functioning	246	61.9 (26.2)	65.9 (24.6)	4.0*	82.5 (18.6)	20.6**
Cognitive functioning	243	66.0 (24.8)	80.5 (23.2)	14.5**	89.7 (15.4)	23.7**
Social functioning	245	65.9 (25.7)	74.2 (28.4)	8.3**	90.6 (18.3)	24.7**
Symptom scales						
Fatigue	240	41.1 (24.8)	36.3 (27.0)	−4.8*	22.0 (21.1)	−19.1**
Nausea/vomiting	240	22.4 (24.6)	10.3 (19.7)	−12.1**	3.0 (10.5)	−19.4**
Pain	247	38.7 (27.5)	30.9 (29.6)	−7.8**	24.9 (26.2)	−13.8**
Dyspnea	240	32.6 (32.3)	20.4 (28.2)	−12.2**	12.8 (22.0)	−19.8**
Insomnia	241	36.5 (29.3)	33.1 (32.6)	−3.4	23.8 (26.7)	−12.7**
Appetite loss	244	32.2 (29.1)	21.7 (31.0)	−10.5**	4.7 (13.7)	−27.5**
Constipation	236	22.6 (26.6)	19.2 (28.8)	−3.4	8.3 (17.3)	−14.3**
Diarrhea	241	19.4 (29.4)	5.8 (15.2)	−13.6**	4.7 (14.7)	−14.7**
Financial difficulties	237	22.2 (25.6)	18.6 (28.6)	−3.6	6.3 (16.8)	−15.9**

All scales have a score range of 0–100. For global health/QoL status and functional scales, a higher score represents a better level of functioning/QoL. For symptom scales, a higher score represents worse symptom severity

*EORTC QLQ-C30* European Organization for Research and Treatment of Cancer Quality of Life Questionnaire–Core 30, *EU5* European Union 5, *HR+/HER2−* hormone receptor-positive/human epidermal growth factor receptor 2-negative, *PRO* patient-reported outcome, *QoL* quality of life, *SD* standard deviation

* *p* < 0.05, ** *p* ≤ 0.001 (Student’s t-test)

^a^Scores from the subset of patients with HR+/HER2− advanced breast cancer currently receiving initial endocrine-based therapy for advanced disease who provided PRO data

^b^Reference values for women (*n* = 1147, all ages) with recurrent/metastatic breast cancer across all lines of treatment taken from Scott et al. [35]

^c^European normative values for women in general population aged 60–69 years from the pooled mean scores of six European normative studies involving 16,151 women and men of all ages, taken from Hinz et al. [36]

**Fig. 1:**
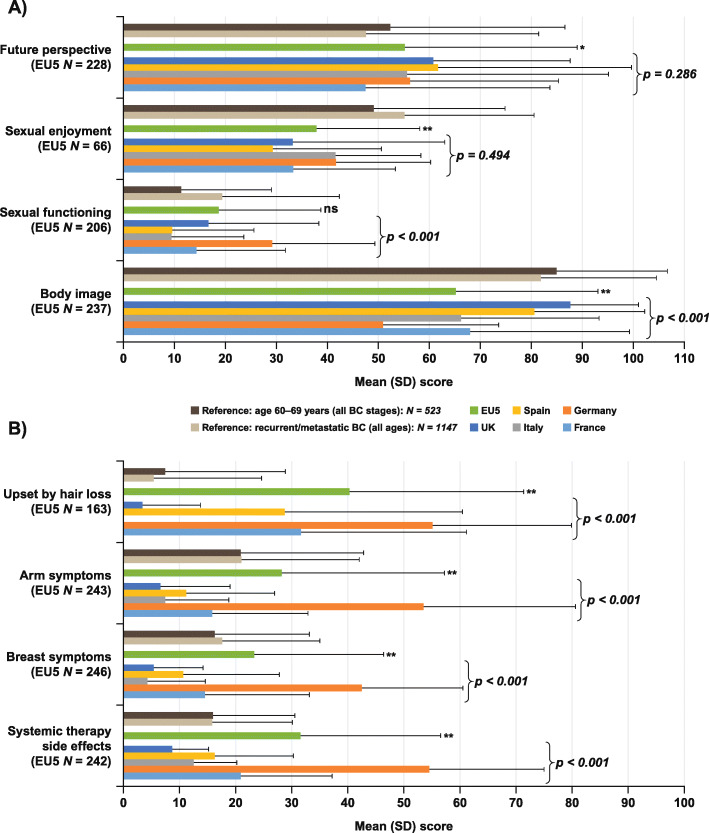
Mean scores for EORTC QLQ-BR23 scales by country and for EU5: **a** functional scales, **b** symptom scales. Each scale has a score range of 0–100, with higher scores representing a better level of functioning or greater symptom severity. Differences between countries *p* < 0.001 (Kruskal–Wallis test) for all scale scores except for sexual enjoyment (*p* = 0.494) and future perspective (*p* = 0.286). Comparisons of mean scores for EU5 vs Reference values for women aged 60–69 years and with recurrent/metastatic breast cancer (taken from Scott et al. [35]) are shown as *p < 0.05, **p < 0.001 (Students t-test). *BC*, breast cancer; *EORTC QLQ-BR23*, European Organization for Research and Treatment of Cancer Quality of Life Questionnaire- 23-item breast cancer-specific module; *EU5*, European Union 5; *NS*, not significant; *UK*, United Kingdom; *SD*, standard deviation

For the EU5, the mean scores for the breast cancer-specific (EORTC QLQ-BR23) functional scales (Fig. 1a) were lowest (more severe) for sexual functioning (18.6) and sexual enjoyment (37.9), followed by future perspective (55.3) and body image (65.2). The scores for body image and sexual functioning differed significantly between countries (*p* < 0.001 for both scales), whereas the future perspective scores were similar between countries. Fewer patients provided a sexual enjoyment score, but the mean scores were similar between countries. Notably, the EU5 mean scores were significantly lower (worse) than the reference values reported for women with recurrent/metastatic breast cancer [35] for future perspective (*p* = 0.002), body image (*p* < 0.001) and sexual enjoyment (*p* < 0.001). The mean scores for the four QLQ-BR23 symptom scales (Fig. 1b) for the EU5 were breast symptoms (23.3), arm symptoms (28.2), systemic therapy side effects (31.5), and upset by hair loss (40.3); all four scores were significantly higher (more severe) than the mean reference values for patients with recurrent/metastatic breast cancer (*p* < 0.001) and differed significantly between countries (*p* < 0.001), with notably higher mean scores in Germany (Fig. 1b).

The mean (SD) EQ-5D-3L health utility index score for the EU5 was 0.69 (0.28), and the mean (SD) EQ-VAS score was 57.98 (21.31) for the subset of patients who provided PRO data. Both scores differed significantly across the countries, as shown in Additional file 5: Table S5, with mean scores ranging from 0.66 to 0.82 for the EQ-5D-3L health utility index score (*p* = 0.011 for the comparison between countries) and 46.41 to 73.08 for the EQ-VAS (*p* < 0.001 for the comparison between countries). The frequency distribution of patient responses for each of the EQ-5D-3L dimensions is presented in Additional file 8: Figure S1. For the EU5, more than half of the patients reported no problems in self-care (61%) and mobility (53%), whereas more than half of the patients reported moderate problems with pain/discomfort (65%) and anxiety/depression (58%). Extreme problems related to anxiety/depression, pain/discomfort, and usual activities were reported by 9, 7, and 5% of EU5 patients, respectively. Notably, 98% of patients in Germany reported some or extreme problems with pain/discomfort compared with 29% in the UK, 56% in Italy, 56% in Spain, and 59% in France (Additional file 8: Figure S1).

### Factors associated with patient HRQoL

From the multiple linear regression analysis, two factors were significantly independently associated with a poorer HRQoL (assessed using the EORTC QLQ-C30 global health/QoL scale score): greater activity impairment due to breast cancer (from the WPAI) and country (specifically, Germany vs. France; Additional file 6: Table S6A). The results of the multi-level model in the sensitivity analysis were consistent with these findings (Additional file 6: Table S6B). The intraclass correlation coefficients for the multi-level model (Additional file 6: Table S6B) indicated that the EORTC QLQ-C30 global health/QoL scale score was only slightly correlated within the same country but was more correlated within the same physician and country. We estimated that physician and country random effects accounted for approximately 50% of the total residual variance.

### Patient-reported pain severity and pain interference

**Fig. 2:**
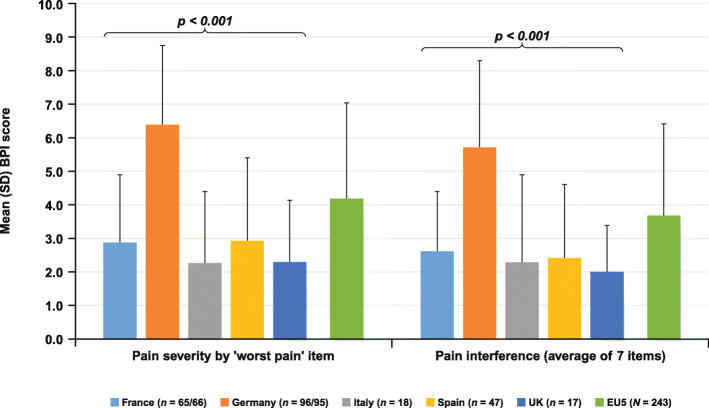
BPI scores for “worst pain” severity and “pain interference” by country and for EU5. Higher scores represent greater pain severity/inference. Differences between countries *p* < 0.001 for both scores (Kruskal–Wallis test). *BPI*, Brief Pain Inventory; *EU5*, European Union 5; *SD*, standard deviation, *UK*, United Kingdom

**Fig. 3:**
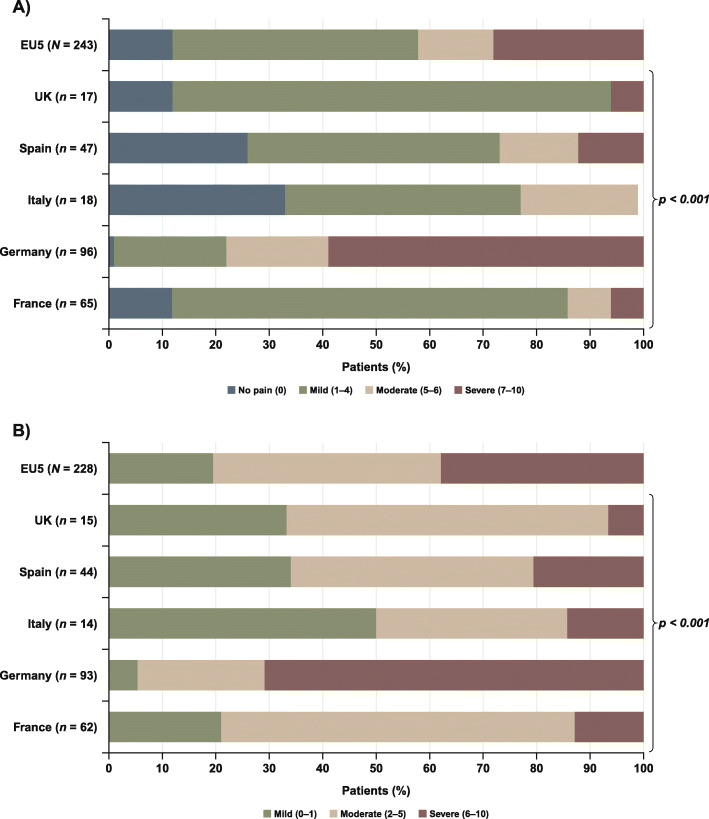
Severity categories for **a** BPI “worst pain” item, **b** average of all seven “pain interference” item scores for all patients who answered the BPI. Differences between countries *p* < 0.001 (Pearson’s Chi-squared test) for both “worst pain” and “pain interference.” *BPI*, Brief Pain Inventory; *EU5*, European Union 5; *UK*, United Kingdom

Mean (SD) BPI scores for the “worst pain” item and the average of the seven “pain interference” items are shown in Fig. 2. The difference between countries was significant (*p* < 0.001) for both pain-related scores, with patients in Germany reporting higher mean scores. When asked to rate the “worst pain” item on the BPI, 46% of the EU5 patients who answered this question rated it as mild, 42% rated it as moderate/severe, and 12% reported no pain (Fig. 3a). In addition, 20% of EU5 patients rated their “pain interference” as mild and 80% as moderate/severe (Fig. 3b); the differences between countries was significant (*p* < 0.001) for both scores. In Germany, 78% of patients reported their “worst pain” as moderate/severe, whereas this ranged from 6% in the UK to 28% in Spain in the other four EU countries. Likewise, 95% of patients in Germany rated their “pain interference” as moderate/severe, compared with 50% in Italy to 79% in France.

### Physician-reported pain among patients

Of the 345 patients (EU5) currently experiencing pain (mild *n* = 259, moderate *n* = 75, severe *n* = 11; physician reported), 4% received no analgesia, 58% non-opioid analgesics, 23% weak opioid analgesics, and 15% strong opioid analgesics (≥75 mg oral morphine equivalents per day).

### Patient-reported work and activity impairment

Because employment rates were low, as expected for this older patient population, no meaningful results for the WPAI questions on absenteeism, presenteeism, and overall work impairment can be presented. WPAI data on daily activity impairment due to breast cancer was available for 246 patients: the mean percentage of total activity impairment for the EU5 was 44%, ranging from 32% in the UK (*n* = 17) to 54% in Germany (*n* = 99). The difference between countries was significant (*p* < 0.001). The WPAI data by country and for the EU5 are summarized in Additional file 7: Table S7.

## Discussion

This single point-in-time real-world survey conducted in 2017 in five European countries shows the unmet needs of women receiving their initial ET-based regimen for HR+/HER2− advanced breast cancer.

The women in our survey who provided PRO data were predominantly older, post-menopausal, not working/retired, had stage IV disease (many with bone [66%] and/or visceral metastases [38%]), were considered by their oncologist to have stable disease (58%) or to be responding to treatment (35%), and had been on their current ET-based regimen for a mean of 6.7 months. The characteristics of this subset of women were similar to the overall EU5 sample with HR+/HER2− advanced breast cancer currently receiving their initial ET-based regimen. They reported a poor overall HRQoL, a high level of symptoms, especially fatigue, pain, and insomnia, and impaired activity and sexual functioning. Unexpectedly, 89% of the PRO subset of women in our survey had advanced disease (stage IIIb/IIIc/IV) at diagnosis. This is much higher than the estimated 28.5% of patients in the comparable de novo metastatic breast cancer cohort in the Epidemiological Strategy and Medical Economics (ESME) research program in France [37]. The reasons for the high percentage of patients with de novo advanced disease in our cohort are unclear, but it may be a methodological issue in that such patients are consulting more frequently in the real-world setting and are therefore more likely to be selected by physicians for the survey than patients who had disease recurrence. Some physicians may have deviated from the protocol and selected more patients with de novo metastatic disease and therefore less involved patient histories to reduce the time burden of completing the PRFs. It may also reflect that improved treatment in the adjuvant setting has caused a shift in stage distribution, such that fewer patients diagnosed with early-stage breast cancer progress to the advanced/metastatic stage [38].

The subset of women providing PRO data in our analyses were considered ET responsive, and 77% were on endocrine-only therapy as initial treatment for advanced disease at the time of the survey, which is consistent with current guideline recommendations [8]. At the time of the survey, only 7% of patients who provided PRO data were receiving a combination of ET plus a CDK4 & 6 inhibitor as their initial therapy for advanced disease. The most probable reason for this is that palbociclib was the only CDK 4 & 6 inhibitor available at the time and had only recently been approved in the EU (clinical trial participants were not included in the DSP), whereas three CDK4 & 6 inhibitors are now available (palbociclib, ribociclib, and abemaciclib). Future studies could determine whether increasing use of such treatments has a positive effect on the HRQoL of patients living with advanced breast cancer.

The development of novel treatments that are effective and well-tolerated, and their use in combination with ET as part of a first-line regimen for advanced disease in patients with HR+/HER2− advanced breast cancer, has led to improvements in progression-free survival [39]. It is important that this also translates into an improved or maintained HRQoL, especially during the time of stable disease. Further research in this area is needed.

In the absence of a cure, HRQoL is an important issue for patients with advanced breast cancer when the goal of treatment is to prolong life while maintaining the quality of survival [8]. HRQoL deteriorates as the disease progresses and can be affected by many patient-, disease-, and treatment-related factors [40, 41]. Not surprisingly, the women with advanced breast cancer in our real-world survey had poorer HRQoL than published general population norms for older women in Europe [36], in agreement with previous studies [42]. However, the mean EORTC QLQ-C30 global health/QoL scale score for our EU5 cohort of women with HR+/HER2− advanced breast cancer (50.9) was lower (worse) than the reference value published in 2008 for women with recurrent/metastatic breast cancer (60.2) [35]. The reason for this difference is unclear and may be due to many factors, including sociodemographic characteristics, disease characteristics, comorbidity, country, care setting, and treatment. Although 58% of the women providing PRO data in our survey were considered by their physician to have stable disease, their mean EORTC QLQ-C30 global health/QoL scale score (50.9) was similar to that reported for a group with progressive disease (52.9) in a real-world study in Canada of postmenopausal women with HR+/HER2− advanced breast cancer receiving first-line treatment [16].

Patients with advanced breast cancer experience a range of symptoms associated with the disease and its treatment that can impact their HRQoL. Fatigue, pain, and insomnia were the EORTC QLQ-C30 symptoms of greatest severity reported by our women with HR+/HER2− advanced breast cancer, consistent with other studies, including real-world surveys and qualitative interviews [12, 43, 44]. In our cohort, BPI “worst pain” was moderate/severe for 42% of patients, and “pain interference” was moderate/severe for 80% of patients. Increased pain severity has been associated with a worse HRQoL or health status in patients with advanced breast cancer [14, 40, 45]. However, patient-reported pain/discomfort was not an independent predictor of HRQoL in our regression analysis despite a high proportion (~ 70%) of the EU5 patients in our analysis reporting some/extreme problems in the EQ-5D-3L domain of pain/discomfort. Although 96% of patients currently experiencing pain reported taking analgesic medication, most of these patients took non-opioid analgesia (58%) or weak opioids (23%). Strong opioid analgesics were taken by only 15% of patients, which is consistent with the low use of strong opioids by patients with advanced breast cancer and bone metastases reported by Cleeland et al. [28]. Taken together, these findings suggest the potential for improved HRQoL with improved pain management. Delaying the onset of pain, reducing pain severity, and reducing pain interference with daily activities are important issues for patients with advanced breast cancer [46]. The patients in Germany had higher and/or more severe pain scores than patients in the other EU countries and this may be because the German patients were more likely to have multiple metastatic sites and lung metastases present at the time of data collection compared with women from EU4 combined.

Bone is the most common site of metastasis in patients with HR+/HER2− advanced breast cancer [7, 47]. Bone metastases were present in 66% of our cohort providing PRO data. Patients with bone metastases report worse pain and poorer HRQoL than those without bone metastases, as shown in previous real-world data from patients with advanced breast cancer in six European countries [48].

A recent analysis of unfavorable prognostic factors in an EU5 and US real-world population of patients with HR+/HER2− advanced breast cancer found that HRQoL was lowest among patients with liver metastases [Davie A, Carter GC, Rider A, Bailey A, Lewis K, Price G, Ostojic H, Ringeisen F, Pivot X: Real-world clinical profile, treatment patterns and outcomes of HR+/HER2− advanced breast cancer patients with unfavorable prognostic factors: data from an international study. submitted]. However, the presence of liver metastases (in 18% of EU5 women providing PRO data) was not an independent predictor of HRQoL in our regression analysis. Analyses of phase III studies aimed at identifying which subgroup of patients may benefit most from a combination of ET plus CDK4 & 6 inhibitor as initial therapy for advanced disease found that patients with indicators of a poor prognosis (liver metastases, progesterone receptor-negative tumors, high-grade tumors, or treatment-free interval < 36 months) benefited most from this combination therapy [49]. Thus, greater use of ET plus a CDK4 & 6 inhibitor as initial therapy for advanced disease in women with HR+/HER2− advanced breast cancer may have the potential to maintain/improve patient HRQoL. However, the poor HRQoL of the women in our survey may be more disease related than treatment related, as they were mostly considered to be stable on treatment.

Our EU5 cohort reported substantial (44%) activity impairment, which is consistent with a previous HR+/HER2− real-world study in the USA and Europe, which reported 57% activity impairment for the total sample [13]. Our regression analysis found that greater activity impairment due to breast cancer was independently associated with worse HRQoL in women with HR+/HER2− advanced breast cancer on their initial ET-based regimen for advanced disease. This agrees with data from the VIRGO observational cohort study in the USA, which reported a significant correlation between percent activity impairment on the WPAI and overall HRQoL in patients with advanced breast cancer [12]. In that study, symptom severity and interference were also strong predictors of activity impairment.

A strength of our study was that we assessed real-world patient outcomes of patients with advanced breast cancer (i.e., women with HR+/HER2− advanced breast cancer receiving an initial ET-based regimen for advanced disease) across five European countries using a variety of well-validated standard PRO instruments, including the frequently used EORTC QLQ-C30. Real-world data complement clinical trial data to help inform patient care [50]. PRO measures are increasingly being used in clinical trials and are broadly applicable in both clinical research and daily clinical practice; they can be used to support decision making by regulators, payers, healthcare providers, and patients [51]. However, the interpretation of EORTC QLQ-C30 scores can be challenging for clinicians [52] and various definitions of a clinically meaningful difference in these scores have been used [53–57]. Nevertheless, for many of the QLQ-C30 and QLQ-BR23 scores, the difference in means between our EU5 sample and the reference value for patients with advanced breast cancer could be considered clinically meaningful. Moreover, there is a need for additional PRO questionnaires for use in patients with advanced breast cancer because the side effects of targeted therapies are not covered by existing instruments, including the EORTC QLQ-BR23 [10]. Symptomatic adverse events from the patient’s perspective can be captured using the PRO version of the Common Terminology Criteria for Adverse Events (PRO-CTCAE) [58], but there are no reports of studies using this tool during routine treatment of patients with breast cancer.

Several limitations of our study must be considered. There may be some selection bias of the physicians and patients included in the study, which may contribute to the high percentage of patients diagnosed with advanced disease. German physicians also had a higher workload than physicians in the other countries. As it was not a mandatory requirement for study participation, only 32% of patients with PRF data completed the PSC forms and provided PRO data. The small sample size of the subset of women reporting PROs should be considered when interpreting the findings. Non-response bias could affect the generalizability of the survey findings and many factors may be associated with non-completion of PRO measures in clinical care [59]. However, the subset of women providing PRO data and the overall sample had similar characteristics. Another factor that may limit the generalizability of the findings is that the data were collected at a single time point for each patient. This also means that causal inferences cannot be made regarding the relationships of interest. For example, although the regression analysis showed that poorer HRQoL (from the EORTC-QLQ-C30) was associated with greater activity impairment (from the WPAI), we do not know the direction of this association. Also, patient-reported symptoms and HRQoL and the factors influencing HRQoL are likely to change over time. As no adjustments for multiple testing were made, care must be taken when interpreting surprising or non-intuitive *p*-values. Although all patients had HR+/HER2− advanced breast cancer and were receiving an initial ET-based regimen for advanced disease, the mean duration of this treatment was only 6.7 months, and this short follow-up time must be considered when interpreting the results. These women were either newly diagnosed with advanced disease or had progressed to advanced disease after having a long disease-free interval (> 12 months) following the cessation of earlier adjuvant treatment. Both situations are likely to have an impact on their HRQoL. Our analyses were primarily descriptive and focused on the EU5; nevertheless, we did find between-country differences in some of the PROs measured. As 40% of the women providing PRO data were from Germany, the data were likely skewed and this must be taken into consideration when interpreting the results. In addition, country (especially Germany) was an independent predictor of HRQoL in the regression analysis. Numerous factors are likely to be associated with the differences between countries, but the higher prevalence of multiple metastatic sites and greater presence of lung metastases at the time of data collection among the German women who provided PRO data compared with the EU4 countries combined is likely to have contributed to their worse pain scores and lower HRQoL. Our study focused on the patient and not the entire burden of advanced breast cancer, which would include the impact on caregivers and on healthcare resource utilization and costs to give a societal perspective. Previous studies have shown that caregivers can experience low HRQoL and high productivity losses [16].

## Conclusions

This real-world survey in 2017 at the start of the change in therapeutic landscape was conducted across the EU5 and demonstrated that women with HR+/HER− advanced breast cancer on an initial ET-based regimen for advanced disease were living with a high level of symptoms, pain, pain interference, activity impairment, and poor overall HRQoL. These findings underscore the need to reduce pain and preserve/improve health status/HRQoL in this patient population. HRQoL is adversely influenced by greater activity impairment, and new treatments that control symptoms, prolong a stable disease state with manageable toxicity, and reduce activity impairment may have a positive effect on the HRQoL of those living with advanced breast cancer. An update to this real-world analysis would be of interest to understand the impact of the CDK4 & 6 inhibitors in this clinical setting.

## Supplementary information

**Additional file 1: Table S1.** Sample sizes for analysis cohort (women with HR+/HER2− advanced breast cancer currently receiving initial ET-based for advanced disease).

**Additional file 2: Table S2.** Physician characteristics.

**Additional file 3: Table S3.** Key characteristics of women with HR+/HER2− advanced breast cancer currently receiving initial endocrine-based therapy for advanced disease.

**Additional file 4: Table S4.** Country-specific patient-reported EORTC QLQ-C30 scores (subset of patients with HR+/HER2− advanced breast cancer currently receiving endocrine-based therapy for advanced disease who provided PRO data).

**Additional file 5: Table S5.** Country-specific patient-reported EQ-5D scores.

**Additional file 6: Table S6A.** Factors associated with HRQoL (EORTC QLQ-C30 global health/QoL scale score) from multiple linear regression analysis for patients with HR+/HER2− advanced breast cancer currently receiving initial endocrine-based therapy for advanced disease who provided PRO data in EU5 (*n* = 252 with at least one data point). **Table S6B.** Sensitivity analysis: Factors associated with EORTC QLQ-C30 global health/ QoL scale score from the 3-level linear mixed-effects model with country and physician as random effects.

**Additional file 7: Table S7.** Work Productivity and Activity Impairment scores.

**Additional file 8: Figure S1.** Frequency distribution of the EQ-5D-3L dimension scores by country and for EU5.

## Data Availability

The dataset supporting the conclusions of this article is included within the article (and its additional files).

## Abbreviations

BPI: Brief Pain Inventory
CDK: Cyclin-dependent kinase
DSP: Disease Specific Programme
ECOG: Eastern Cooperative Oncology Group
EORTC QLQ-C30: European Organisation for Research and Treatment of Cancer Quality of Life Questionnaire
EQ-5D-3L: 3-level EuroQol-5 Dimension instrument
EQ-VAS: EuroQol Visual Analogue Scale
ESO–ESMO: European School of Oncology–European Society for Medical Oncology
ESME: Epidemiological Strategy and Medical Economics
ET: Endocrine therapy
EU5: European Union 5
FEKI: Freiburg Ethics Commission International
HER2+/−: Human epidermal growth factor receptor 2 positive/negative
HR+: Hormone receptor positive
HRQoL: Health-related quality of life
ICC/ESOMAR: International Chamber of Commerce/European Society for Opinion and Marketing Research
IQR: Interquartile range
mTOR: Mammalian target of rapamycin
PRF: Patient record form
PRO: Patient-reported outcome
PRO-CTCAE: PRO version of the Common Terminology Criteria for Adverse Events
PS: Performance status
PSC: Patient self-completion
QLQ-BR23: Breast cancer-specific module of the EORTC QLQ-C30
SD: Standard deviation
UK: United Kingdom
US: United States
WPAI:SHP: Work Productivity and Activity Impairment Questionnaire: Specific Health Problem version
