# Supplementary material for: Real-world patient-reported outcomes of women receiving initial endocrine-based therapy for HR+/HER2− advanced breast cancer in five European countries
Source: BMC Cancer. 2020 Sep 7;20:855. doi: 10.1186/s12885-020-07294-2 (PMC7487722; doi:10.1186/s12885-020-07294-2)
Supplement: Supplementary file 1 — Additional file 1: Table S1. Sample sizes for analysis cohort (women with HR+/HER2− advanced breast cancer currently receiving initial ET-based for advanced disease). [file 12885_2020_7294_MOESM1_ESM.docx]

**Additional file 1**

**Table S1** Sample sizes for analysis cohort (women with HR+/HER2− advanced breast cancer currently receiving initial ET-based regimen for advanced disease)

| **Sample** | **France** | **Germany** | **Italy** | **Spain** | **UK** | **Total (EU5)** |
| --- | --- | --- | --- | --- | --- | --- |
| Patients with a PRF completed by their physician | 137 | 197 | 120 | 203 | 124 | 781 |
| Patients who completed a PSC | 69 | 100 | 18 | 48 | 17 | 252 |
| Physicians | 50 | 56 | 41 | 45 | 34 | 226 |

ET, endocrine therapy; EU5, European Union 5; HR+/HER2−, hormone receptor positive/human epidermal growth factor receptor 2 negative; PRF, patient record form; PSC, patient self-completion form; UK, United Kingdom
