# Supplementary material for: Real-world patient-reported outcomes of women receiving initial endocrine-based therapy for HR+/HER2− advanced breast cancer in five European countries
Source: BMC Cancer. 2020 Sep 7;20:855. doi: 10.1186/s12885-020-07294-2 (PMC7487722; doi:10.1186/s12885-020-07294-2)
Supplement: Supplementary file 2 — Additional file 2: Table S2. Physician characteristics. [file 12885_2020_7294_MOESM2_ESM.docx]

**Additional file 2**

**Table S2** Physician characteristics^a^

| **Characteristics** | **France**  **(*N* = 50)** | **Germany**  **(*N* = 56)** | **Italy**  **(*N* = 41)** | **Spain**  **(*N* = 45)** | **UK**  **(*N* = 34)** | **EU5**  **(*N* = 226)** |
| --- | --- | --- | --- | --- | --- | --- |
| Sex, *n* (%) male | 27 (54) | 35 (62) | 21 (51) | 19 (42) | 21 (62) | 123 (54) |
| Clinical setting, %^b^, mean (SD) |  |  |  |  |  |  |
| Comprehensive cancer center | 28.2 (44.5) | 32.0 (46.6) | 42.9 (49.3) | 10.2 (27.8) | 62.6 (40.3) | 33.4 (45.2) |
| Public hospital | 58.7 (49.0) | 37.9 (48.1) | 50.5 (47.7) | 76.2 (39.2) | 29.4 (36.1) | 51.1 (47.3) |
| Private hospital | 7.1 (24.7) | 0.4 (2.7) | 0.5 (3.1) | 4.9 (17.3) | 2.7 (4.9) | 3.1(14.2) |
| Public office | 2.0 (14.1) | 29.6 (45.1) | 5.5 (18.2) | 4.6 (19.1) | 4.6(12.6) | 10.4 (28.6) |
| Private office | 4.0 (19.8) | 0.2 (1.3) | 0.5 (3.1) | 4.1 (15.0) | 0.7 (3.5) | 1.9 (11.7) |
| Hospital type^c^, % | (*n* = 33) | (*n* = 22) | (*n* = 22) | (*n* = 39) | (*n* = 11) | (*n* = 127) |
| University | 48 | 9 | 14 | 82 | 82 | 49 |
| General | 39 | 68 | 73 | 8 | 18 | 39 |
| Community | 9 | 23 | 14 | 5 | 0 | 10 |
| Other | 3 | 0 | 0 | 5 | 0 | 2 |
| Academic/teaching duties, %^d^  Mean (SD) [median] | 14.9 (17.8) [10] | 2.4 (4.6) [0] | 5.3 (8.0) [0] | 14.7 (17.8) [10] | 21.9 (29.2) [10] | 11.0 (17.9) [10] |
| Clinical trial experience^e^, *n* (%) yes | 48 (96) | 29 (52) | 36 (88) | 44 (98) | 33 (97) | 190 (84) |
| General workload |  |  |  |  |  |  |
| Mean number of patients with any condition managed by physician^f^ | 402.6 | 1572.2 | 211.0 | 400.6 | 322.9 | 643.3 |
| Patients with breast cancer, % | 31 | 14 | 45 | 47 | 56 | 25 |
| Breast cancer patients with advanced breast cancer, % | 47 | 45 | 50 | 30 | 50 | 43 |

^a^Physicians with patients in the analysis cohort

^b^Proportion of ambulatory care/outpatients seen in each practice setting

^c^Physicians who spent the majority of their time in hospital

^d^Proportion of time spent on academic/teaching duties

^e^Clinical trial experience in breast cancer

^f^Mean number of patients managed by physicians based on the number of physicians. This was a general question with no timescale

EU5, European Union 5; SD, standard deviation; UK, United Kingdom
