# Supplementary material for: Real-world patient-reported outcomes of women receiving initial endocrine-based therapy for HR+/HER2− advanced breast cancer in five European countries
Source: BMC Cancer. 2020 Sep 7;20:855. doi: 10.1186/s12885-020-07294-2 (PMC7487722; doi:10.1186/s12885-020-07294-2)
Supplement: Supplementary file 3 — Additional file 3: Table S3. Key characteristics of women with HR+/HER2− advanced breast cancer currently receiving initial endocrine-based therapy for advanced disease. [file 12885_2020_7294_MOESM3_ESM.docx]

**Additional file 3**

**Table S3** Key characteristics of women with HR+/HER2− advanced breast cancer currently receiving initial endocrine-based therapy for advanced disease: overall patient sample (*n* = 781)

| **Characteristics** | **France**  **(*N* = 137)** | **Germany**  **(*N* = 197)** | **Italy**  **(*N* = 120)** | **Spain**  **(*N* = 203)** | **UK**  **(*N* = 124)** | **EU5**  **(*N* = 781)** | **Comparison between EU5 countries**  ***p*-value [test used]** | **Germany vs. EU4**  ***p*-value [test used]** |
| --- | --- | --- | --- | --- | --- | --- | --- | --- |
| Age,^a^ years, mean (SD) | 69.4 (10.6) | 60.5 (11.8) | 67.5 (12.3) | 70.7 (11.7) | 70.8 (10.2) | 67.4 (12.1) | <0.001 [AN] | 0.000 [TT] |
| BMI, kg/m^2^, mean (SD) | 24.4 (3.7) | 24.1 (3.5) | 23.3 (3.5) | 25.7 (3.4) | 25.0 (3.4) | 24.6 (3.6) | <0.001 [AN] | 0.021 [TT] |
| Ethnicity, *n* (%)  White/Caucasian  Other^b^ | 123 (90)  14 (10) | 178 (90)  19 (10) | 120 (100)  0 | 192 (95)  11 (5) | 114 (92)  10 (8) | 727 (93)  54 (7) | 0.006 [CH] | 0.103 [CH] |
| Employment status, *n* (%)  Retired/homemaker/unemployed  Employed FT/PT  Long-term sick leave (FT/PT) | (*n* = 133)  118 (89)  10 (7)  5 (4) | (*n* = 195)  139 (71)  38 (20)  18 (9) | (*n* = 116)  97 (84)  19 (16)  0 | (*n* = 190)  161 (85)  20 (11)  9 (5) | (*n* = 108)  99 (92)  8 (7)  1 (1) | (*n* = 742)  614 (83)  95 (13)  33 (4) | <0.001 [CH] | <0.001 [CH] |
| Current^c^ ECOG status^d^, *n* (%)  0  1  2  3‒5 | (*n* = 136)  27 (20)  84 (62)  19 (14)  6 (4) | (*n* = 196)  88 (45)  79 (40)  25 (13)  4 (2) | (*n* = 120)  51 (43)  52 (43)  14 (12)  3 (3) | (*n* = 203)  67 (33)  99 (49)  34 (17)  3 (1) | (*n* = 124)  29 (23)  64 (52)  26 (21)  5 (4) | (*n* = 779)  262 (34)  378 (49)  118 (15)  21 (3) | <0.001 [CH] | 0.002 [CH] |
| Current^c^ menopausal status, *n* (%)  Pre-/perimenopausal  Postmenopausal^e^ | 7 (5)  130 (95) | 15 (8)  182 (92) | 11 (9)  109 (91) | 14 (7)  189 (93) | 5 (4)  119 (96) | 52 (7)  729 (93) | 0.489 [CH] | 0.513 [FE] |
| Number of current^c^ metastases sites^f^, n (%)  1  2  3  4‒5 | (*n* = 132)  92 (70)  30 (23)  9 (7)  1 (1) | (*n* = 126)  68 (54)  41 (33)  13 (10)  4 (3) | (*n* = 112)  79 (71)  28 (25)  5 (5)  0 | (*n* = 198)  103 (52)  81 (41)  14 (7)  0 | (*n* = 119)  60 (50)  48 (40)  10 (8)  1 (1) | (*n* = 687)  402 (59)  228 (33)  51 (7)  6 (1) | 0.002 [AN] | 0.008 [TT] |
| Site of current^c^ metastases^f^, *n* (%)  Bone only  Bone  Liver  Lung  Lymph node involvement  Visceral^g^  Other (including brain & pancreas) | (*n* = 132)  61 (46)  89 (67)  24 (18)  32 (24)  29 (22)  54 (41)  8 (6) | (*n* = 126)  15 (12)  39 (31)  30 (24)  37 (29)  93 (74)  57 (45)  7 (6) | (*n* = 112)  53 (47)  80 (71)  19 (17)  21 (19)  28 (25)  37 (33)  9 (8) | (*n* = 198)  77 (39)  145 (73)  14 (7)  72 (36)  67 (34)  85 (43)  9 (5) | (*n* = 119)  37 (31)  77 (65)  12 (10)  41 (35)  57 (48)  49 (41)  3 (3) | (*n* = 687)  243 (35)  430 (63)  99 (14)  203 (30)  274 (40)  282 (41)  29 (4) | [CH]  <0.001  <0.001  <0.001  0.008  <0.001  0.383  0.379 | [FE]  <0.001  <0.001  0.002  1  <0.001  0.317  0.460 |
| Breast cancer status at diagnosis, *n* (%)  Stage IIb/IIIc/IV  Stage I/II/IIIa | 115 (84)  22 (16) | 192 (98)  5 (3) | 102 (85)  18 (15) | 178 (88)  25 (12) | 117 (94)  7 (6) | 704 (90)  77 (10) | <0.001 [CH] | <0.001 [FE] |
| Current^c^ disease status^h^, *n* (%)  Stable  Tumor responding to treatment  Progressing | 73 (53)  55 (40)  9 (7) | 148 (75)  37 (19)  12 (6) | 87 (73)  30 (25)  3 (3) | 118 (58)  79 (39)  6 (3) | 66 (53)  55 (44)  3 (2) | 492 (63)  256 (33)  33 (4) | <0.001 [CH] | <0.001 [CH] |
| Current^c^ treatment class^i^, *n* (%)  Endocrine only  Endocrine + targeted^j^  Endocrine + chemotherapy  Endocrine + chemotherapy + targeted  Endocrine + targeted + other (unspecified)  Endocrine + other (unspecified) | 102 (74)  18 (13)  6 (4)  10 (7)  0  1 (1) | 158 (80)  22 (11)  12 (6)  5 (3)  0  0 | 95 (79)  17 (14)  4 (3)  0  0  4 (3) | 188 (93)  9 (4)  4 (2)  0  0  2 (1) | 111 (90)  5 (4)  4 (3)  0  1 (1)  3 (2) | 654 (84)  71 (9)  30 (4)  15 (2)  1 (<1)  10 (1) | <0.001 [CH] | 0.096 [CH] |
| Current ET-based regimen duration^k^ (ongoing), months  Mean (SD)  Median  IQR | (*n* = 135)  5.4 (6.8)  3.3  1.7–6.6 | (*n* = 196)  8.5 (9.6)  5.5  2.5–12.2 | (*n* = 120)  4.6 (5.9)  2.7  1.6–5.1 | (*n* = 203)  5.7 (7.2)  2.8  1.7–7.2 | (*n* = 123)  7.1 (20.2)  2.9  1.7–8.3 | (*n* = 777)  6.4 (10.8)  3.5  1.8–7.8 | 0.009 [AN] | 0.002 [TT] |
| TFI^l^, months  Mean (SD)  Median  IQR | (*n* = 22)  42.5 (36.2)  20.7  18.8–56.9 | (*n* = 5)  57.8 (25.1)  58.0  39.5–73.3 | (*n* = 18)  72.5 (66.3)  48.0  25.0–128.9 | (*n* = 25)  47.1 (47.8)  35.4  19.8–57.1 | (*n* = 7)  38.3 (32.0)  27.4  16.8–54.0 | (*n* = 77)  51.6 (48.3)  36.3  19.0–64.5 | 0.291 [AN] | 0.771 [TT] |

Data taken from PRF

^a^Patients reported to be ≥90 years of age were assumed to be 90 years of age for the purposes of this calculation

^b^Includes Afro-Caribbean, Hispanic/Latino, mixed race, Asian-other, Asian-Indian subcontinent, Middle Eastern and Chinese

^c^Current = time of data collection

^d^0 = Fully active, able to carry on all pre-disease performance without restriction; 1 = Restricted in physically strenuous activity but ambulatory and able to carry out light work; 2 = Ambulatory and capable of all self-care but unable to carry out work activities. Up and about more than 50% of waking; 3 = Capable of only limited self-care, confined to bed or chair more than 50% of waking hours; 4 = Completely disabled. Cannot carry out any self-care. Confined to bed or chair; two patients were not assessed

^e^Including natural, medically induced ovary suppression and ablation

^f^Based on patients currently at stage IV

^g^Visceral metastases = presence of brain, liver, lungs, pancreas, and other (including pleural) metastases

^h^Physician reported

^i^Simultaneous treatments

^j^Targeted therapy for any patient included palbociclib (*n* = 36), everolimus (*n* = 34), bevacizumab (*n* = 15), and other (*n* = 3)

^k^All patients were currently still on first-line therapy; duration does not reflect how long patients stay on a first-line regimen

^l^Treatment-free interval between adjuvant therapy and first-line advanced ET for the patients with stage I/II/IIIa at diagnosis

AN, analysis of variance; BMI, body mass index; CH, Pearson’s Chi-squared test; ECOG, Eastern Cooperative Oncology Group; ET, endocrine therapy; EU5, European Union 5; EU4, European Union 4 (France, Italy, Spain and UK); FE, Fisher’s exact test; FT, full time; HER2+, human epidermal growth factor receptor 2-positive; HR+/HER2−, hormone receptor-positive/human epidermal growth factor receptor 2-negative; IQR, interquartile range; PRF, patient record form; PT, part time; SD, standard deviation; TFI, treatment-free interval; TT, Students t-test; UK, United Kingdom
