# Supplementary material for: Real-world patient-reported outcomes of women receiving initial endocrine-based therapy for HR+/HER2− advanced breast cancer in five European countries
Source: BMC Cancer. 2020 Sep 7;20:855. doi: 10.1186/s12885-020-07294-2 (PMC7487722; doi:10.1186/s12885-020-07294-2)
Supplement: Supplementary file 4 — Additional file 4: Table S4. Country-specific patient-reported EORTC QLQ-C30 scores (subset of patients with HR+/HER2− advanced breast cancer currently receiving endocrine-based therapy for advanced disease who provided PRO data). [file 12885_2020_7294_MOESM4_ESM.docx]

**Additional file 4**

**Table S4** Country-specific patient-reported EORTC QLQ-C30 scores (subset of patients who provided PRO data)^a^

| **Scales** | **France** | | **Germany** | | **Italy** | | **Spain** | | **UK** | | **EU4** | |
| --- | --- | --- | --- | --- | --- | --- | --- | --- | --- | --- | --- | --- |
|  | ***n*** | **Mean (SD)** | ***n*** | **Mean (SD)** | ***n*** | **Mean (SD)** | ***n*** | **Mean (SD)** | ***n*** | **Mean (SD)** | ***n*** | **Mean (SD)** |
| Global health/QoL scale | 66 | 58.0 (19.0) | 100 | 35.8 (22.4) | 18 | 61.6 (17.9) | 48 | 62.7 (25.0) | 17 | 67.2 (16.3) | 149 | 61.0 (20.8) |
| *Functional scales* |  |  |  |  |  |  |  |  |  |  |  |  |
| Physical functioning | 67 | 71.2 (19.9) | 96 | 46.0 (19.7) | 18 | 80.4 (18.8) | 47 | 76.0 (21.7) | 17 | 78.8 (21.4) | 149 | 74.7 (20.6) |
| Role functioning | 67 | 65.4 (25.0) | 95 | 43.5 (22.3) | 18 | 78.7 (22.7) | 47 | 73.8 (24.5) | 17 | 71.6 (24.8) | 149 | 70.4 (24.8) |
| Emotional functioning | 67 | 68.7 (23.8) | 96 | 47.9 (23.3) | 18 | 67.6 (23.2) | 48 | 73.8 (26.4) | 17 | 75.5 (17.0) | 150 | 70.9 (23.9) |
| Cognitive functioning | 67 | 71.1 (21.0) | 93 | 49.8 (22.0) | 18 | 82.4 (21.0) | 48 | 78.5 (20.3) | 17 | 82.4 (17.1) | 150 | 76.1 (20.7) |
| Social functioning | 65 | 72.6 (23.1) | 97 | 51.0 (22.5) | 18 | 82.4 (23.9) | 48 | 76.4 (23.8) | 17 | 78.4 (17.4) | 148 | 75.7 (22.9) |
| *Symptom scales* |  |  |  |  |  |  |  |  |  |  |  |  |
| Fatigue | 67 | 36.8 (23.9) | 91 | 53.2 (21.0) | 18 | 35.2 (23.9) | 47 | 28.8 (26.1) | 17 | 33.3 (19.2) | 149 | 33.7 (24.2) |
| Nausea/vomiting | 67 | 16.4 (19.1) | 91 | 41.9 (23.7) | 18 | 2.8 (6.4) | 47 | 6.7 (12.4) | 17 | 5.9 (11.7) | 149 | 10.5 (16.1) |
| Pain | 67 | 29.6 (22.4) | 97 | 57.2 (22.8) | 18 | 17.6 (23.9) | 48 | 27.8 (24.6) | 17 | 21.6 (21.1) | 150 | 26.7 (23.3) |
| Dyspnea | 67 | 23.4 (23.9) | 91 | 58.6 (28.7) | 18 | 11.1 (19.8) | 47 | 13.5 (22.7) | 17 | 5.9 (13.1) | 149 | 16.8 (22.8) |
| Insomnia | 67 | 29.4 (25.0) | 92 | 55.4 (26.3) | 18 | 24.1 (22.3) | 47 | 22.0 (26.3) | 17 | 15.7 (17.1) | 149 | 24.8 (24.6) |
| Appetite loss | 67 | 25.4 (22.5) | 95 | 48.8 (31.1) | 18 | 13.0 (20.3) | 47 | 20.6 (23.6) | 17 | 19.6 (16.9) | 149 | 21.7 (22.2) |
| Constipation | 66 | 22.2 (26.4) | 88 | 33.7 (27.9) | 18 | 1.9 (7.9) | 47 | 14.9 (20.6) | 17 | 9.8 (22.9) | 148 | 16.0 (23.5) |
| Diarrhea | 66 | 11.1 (18.8) | 92 | 39.5 (35.3) | 18 | 5.6 (12.8) | 48 | 3.5 (10.3) | 17 | 2.0 (8.1) | 149 | 6.9 (15.1) |
| Financial difficulties | 66 | 15.2 (21.2) | 88 | 41.3 (24.8) | 18 | 3.7 (10.8) | 48 | 9.0 (16.5) | 17 | 7.8 (14.6) | 149 | 11.0 (18.4) |

^a^Scores from the subset of patients with HR+/HER2− advanced breast cancer currently receiving initial endocrine-based therapy for advanced disease who provided PRO data

All scales have a score range of 0‒100. For global health/QoL scale and functional scale, a higher score represents a better level of functioning/QoL. For symptom scales, a higher score represents worse symptom severity

For all scale scores, between-country comparisons were *p* < 0.001 (Kruskall–Wallis test), comparisons for EU4 vs Germany were *p* < 0.001 (Mann-Whitney test), and comparisons for Germany vs Reference values from women with advanced breast cancer taken from Scott et al. [35] were *p* < 0.001 (Student’s t-test)

EORTC QLQ-C30, European Organization for Research and Treatment of Cancer Quality of Life Questionnaire–Core 30; EU4, European Union 4 (France, Italy, Spain, UK); HR+/HER2−, hormone receptor positive/human epidermal growth factor receptor 2 negative; PRO, patient-reported outcome; QoL, quality of life; SD, standard deviation; UK, United Kingdom
