# Supplementary material for: Real-world patient-reported outcomes of women receiving initial endocrine-based therapy for HR+/HER2− advanced breast cancer in five European countries
Source: BMC Cancer. 2020 Sep 7;20:855. doi: 10.1186/s12885-020-07294-2 (PMC7487722; doi:10.1186/s12885-020-07294-2)
Supplement: Supplementary file 5 — Additional file 5: Table S5. Country-specific patient-reported EQ-5D scores. [file 12885_2020_7294_MOESM5_ESM.docx]

**Additional file 5**

**Table S5** Country-specific patient-reported EQ-5D scores (subset of patients who provided PRO data)^a^

| Country | **EQ-5D-3L HUI** | | | **EQ-VAS** | | |
| --- | --- | --- | --- | --- | --- | --- |
|  | ***n*** | **Mean (SD)** | **Median (IQR)** | ***n*** | **Mean (SD)** | **Median (IQR)** |
| France | 69 | 0.66 (0.32) | 0.73 (0.37–0.91) | 69 | 60.57 (18.05) | 60.00 (49.50–75.00) |
| Germany | 98 | 0.66 (0.24) | 0.70 (0.70–0.80) | 100 | 46.41 (20.58) | 40.00 (30.00–65.00) |
| Italy | 18 | 0.82 (0.14) | 0.86 (0.73–0.91) | 18 | 59.83 (13.94) | 59.00 (50.00–70.00) |
| Spain | 48 | 0.72 (0.34) | 0.83 (0.53–1.00) | 48 | 73.08 (18.38) | 70.00 (60.00–90.00) |
| UK | 17 | 0.79 (0.17) | 0.85 (0.70–0.85) | 17 | 70.88 (13.80) | 75.00 (62.00–81.50) |
| EU5 | 250 | 0.69 (0.28) | 0.79 (0.58–0.89) | 252 | 57.98 (21.31) | 60.00 (40.00–75.00) |
| *p*-value |  | 0.011 | |  | <0.001 | |

^a^Scores from the subset of patients with HR+/HER2− advanced breast cancer currently receiving initial endocrine-based therapy for advanced disease who provided PRO data. A higher score represents a better health status

*P*-value for between-country comparison (Kruskal–Wallis test)

EQ-5D-3L HUI, EuroQoL 5-dimension 3-level questionnaire health utility index; EQ-VAS, EuroQoL visual analogue scale; EU5, European Union 5; HR+/HER2−, hormone receptor positive/ human epidermal growth factor receptor 2 negative; IQR, interquartile range; PRO, patient-reported outcome; SD, standard deviation; UK, United Kingdom
