# Supplementary material for: Real-world patient-reported outcomes of women receiving initial endocrine-based therapy for HR+/HER2− advanced breast cancer in five European countries
Source: BMC Cancer. 2020 Sep 7;20:855. doi: 10.1186/s12885-020-07294-2 (PMC7487722; doi:10.1186/s12885-020-07294-2)
Supplement: Supplementary file 6 — Additional file 6: Table S6A. Factors associated with HRQoL (EORTC QLQ-C30 global health/QoL scale score) from multiple linear regression analysis for patients with HR+/HER2− advanced breast cancer currently receiving initial endocrine-based therapy for advanced disease who provided PRO data in EU5 (n = 252 with at least one data point). Table S6B. Sensitivity analysis: Factors associated with EORTC QLQ-C30 global health/ QoL scale score from the 3-level linear mixed-effects model with country and physician as random effects. [file 12885_2020_7294_MOESM6_ESM.docx]

**Additional file 6**

**Table S6A** Factors associated with HRQoL (EORTC QLQ-C30 global health/QoL scale score) from multiple linear regression analysis^a^

| Variable (R^2^ = 0.56) | Coefficient^b^ | SE of estimate^c^ | 95% CI | *P* value |
| --- | --- | --- | --- | --- |
| Age (years) | 0.096 | 0.109 | −0.120 to 0.313 | 0.379 |
| Number of comorbidities | −1.191 | 0.864 | −2.906 to 0.524 | 0.171 |
| ECOG score |  |  |  |  |
| 0 (reference group) | 0 |  |  |  |
| 1 | −0.227 | 3.128 | −6.438 to 5.984 | 0.942 |
| 2–4 | −6.005 | 4.582 | −15.104 to 3.095 | 0.193 |
| Disease status |  |  |  |  |
| Responding (reference group) | 0 |  |  |  |
| Stable | 1.933 | 2.727 | −3.482 to 7.348 | 0.480 |
| Progressing | −3.953 | 5.373 | −14.623 to 6.717 | 0.464 |
| Liver metastases |  |  |  |  |
| Absent (reference group) | 0 |  |  |  |
| Present | −1.380 | 3.276 | −7.886 to 5.125 | 0.674 |
| Number of current metastases (excluding liver metastases) | −0.581 | 1.754 | −4.064 to 2.902 | 0.741 |
| Pain^d^ |  |  |  |  |
| No pain/discomfort (reference group) | 0 |  |  |  |
| Pain/discomfort | −1.635 | 3.156 | −7.903 to 4.632 | 0.606 |
| Ongoing treatment duration (weeks) | 0.037 | 0.024 | −0.011 to 0.086 | 0.130 |
| Activity impairment^e^ (% impairment) | −0.521 | 0.072 | −0.663 to −0.378 | 0.000 |
| Country |  |  |  |  |
| France (reference group) | 0 |  |  |  |
| Germany | −15.060 | 4.319 | −23.636 to −6.485 | 0.001 |
| Italy | −1.650 | 4.440 | −10.467 to 7.168 | 0.711 |
| Spain | 0.337 | 4.226 | −8.054 to 8.729 | 0.937 |
| UK | 2.438 | 5.214 | −7.915 to 12.791 | 0.641 |

^a^Patients with HR+/HER2− advanced breast cancer currently receiving initial endocrine-based therapy for advanced disease who provided PRO data in EU5 (*n* = 252 with at least one data point)

^b^For the numerical variables (age, number of comorbidities, number of current metastases, ongoing treatment duration, and activity impairment), the coefficient represents a 1-unit change

^c^SEs were adjusted to allow for intragroup correlation within reporting physician

^d^Based on the patient-reported response to EQ-5D question on pain/discomfort

^e^Total activity impairment score on WPAI

CI, confidence interval; ECOG, Eastern Cooperative Oncology Group; EORTC QLQ-C30, European Organization for Research and Treatment of Cancer Quality of Life Questionnaire–Core 30; EQ-5D, EuroQol 5-dimension questionnaire; EU5, European Union 5; HR+/HER2−, hormone receptor positive/human epidermal growth factor receptor 2 negative; HRQoL, health-related quality of life; PRF, patient record form; PRO, patient-reported outcome; QoL, quality of life; SE, standard error; UK, United Kingdom; WPAI, Work Productivity and Activity Impairment questionnaire

**Table S6B** Sensitivity analysis: Factors associated with EORTC QLQ-C30 global health/ QoL scale score from the 3-level linear mixed-effects model with country and physician as random effects

|  | Coefficient | SE of estimate | 95% CI | *P* value |
| --- | --- | --- | --- | --- |
| Age (years) | 0.158 | 0.097 | −0.033 to 0.348 | 0.105 |
| Number of comorbidities | −1.326 | 0.784 | −2.862 to 0.211 | 0.091 |
| ECOG score |  |  |  |  |
| 0 (reference group) | 0 |  |  |  |
| 1 | −0.556 | 2.411 | −5.281 to 4.168 | 0.817 |
| 2–4 | −7.396 | 3.451 | −14.161 to −0.632 | 0.032 |
| Disease status |  |  |  |  |
| Responding (reference group) | 0 |  |  |  |
| Stable | 0.161 | 2.238 | −4.226 to 4.547 | 0.943 |
| Progressing | −3.309 | 4.715 | −12.549 to 5.932 | 0.483 |
| Liver metastases |  |  |  |  |
| Absent (reference group) | 0 |  |  |  |
| Present | −3.146 | 2.999 | −9.023 to 2.732 | 0.294 |
| Number of current metastases (excluding liver metastases) | −0.032 | 1.348 | −2.674 to 2.610 | 0.981 |
| Pain |  |  |  |  |
| No pain/discomfort (reference group) | 0 |  |  |  |
| Pain/discomfort | −5.090 | 2.723 | −10.426 to 0.247 | 0.062 |
| Ongoing treatment duration (weeks) | 0.020 | 0.032 | −0.042 to 0.083 | 0.525 |
| Activity impairment (% impairment) | −0.386 | 0.054 | −0.491 to −0.281 | 0.000 |

| **Level** | **Intraclass correlation coefficient** | **SE** | **95% CI** |
| --- | --- | --- | --- |
| Country | 0.111 | 0.083 | 0.024 to 0.394 |
| Physician/country | 0.499 | 0.082 | 0.343 to 0.655 |

CI, confidence interval; ECOG, Eastern Cooperative Oncology Group; EORTC QLQ-C30, European Organization for Research and Treatment of Cancer Quality of Life Questionnaire–Core 30; QoL, quality of life; SE, standard error
