# Supplementary material for: Real-world patient-reported outcomes of women receiving initial endocrine-based therapy for HR+/HER2− advanced breast cancer in five European countries
Source: BMC Cancer. 2020 Sep 7;20:855. doi: 10.1186/s12885-020-07294-2 (PMC7487722; doi:10.1186/s12885-020-07294-2)
Supplement: Supplementary file 7 — Additional file 7: Table S7. Work Productivity and Activity Impairment scores. [file 12885_2020_7294_MOESM7_ESM.docx]

**Additional file 7**

**Table S7** Work Productivity and Activity Impairment scores

| **WPAI score** | **France** | | **Germany** | | **Italy** | | **Spain** | | **UK** | | **EU5** | |
| --- | --- | --- | --- | --- | --- | --- | --- | --- | --- | --- | --- | --- |
|  | ***n*** | **Mean (SD)** | ***n*** | **Mean (SD)** | ***n*** | **Mean (SD)** | ***n*** | **Mean (SD)** | ***n*** | **Mean (SD)** | ***n*** | **Mean (SD)** |
| Absenteeism | 2 | 14.3 (20.2) | 7 | 21.4 (39.3) | 2 | 22.5 (3.5) | 2 | 8.3 (11.8) | 1 | 0 | 14 | 17.2 (28.4) |
| Presenteeism | 4 | 45.0 (23.8) | 17 | 54.1 (23.7) | 2 | 45.0 (7.1) | 3 | 10.0 (10.0) | 1 | 10 (–) | 27 | 45.6 (25.8) |
| Overall work impairment | 2 | 70.7 (1.0) | 7 | 55.0 (29.3) | 2 | 57.3 (7.4) | 2 | 18.3 (2.4) | 1 | 10 (–) | 14 | 49.1 (27.7) |
| Total activity impairment | 64 | 39.8 (23.6) | 99 | 54.4 (20.3) | 18 | 33.3 (25.2) | 48 | 34.2 (25.8) | 17 | 31.8 (19.8) | 246 | 43.6 (24.3)* |

Scores are expressed as percentages, with a higher percentage indicating greater work/activity impairment

EU5, European Union 5; SD, standard deviation; UK, United Kingdom; WPAI, work productivity and activity impairment

**p* < 0.001 for comparison between countries
